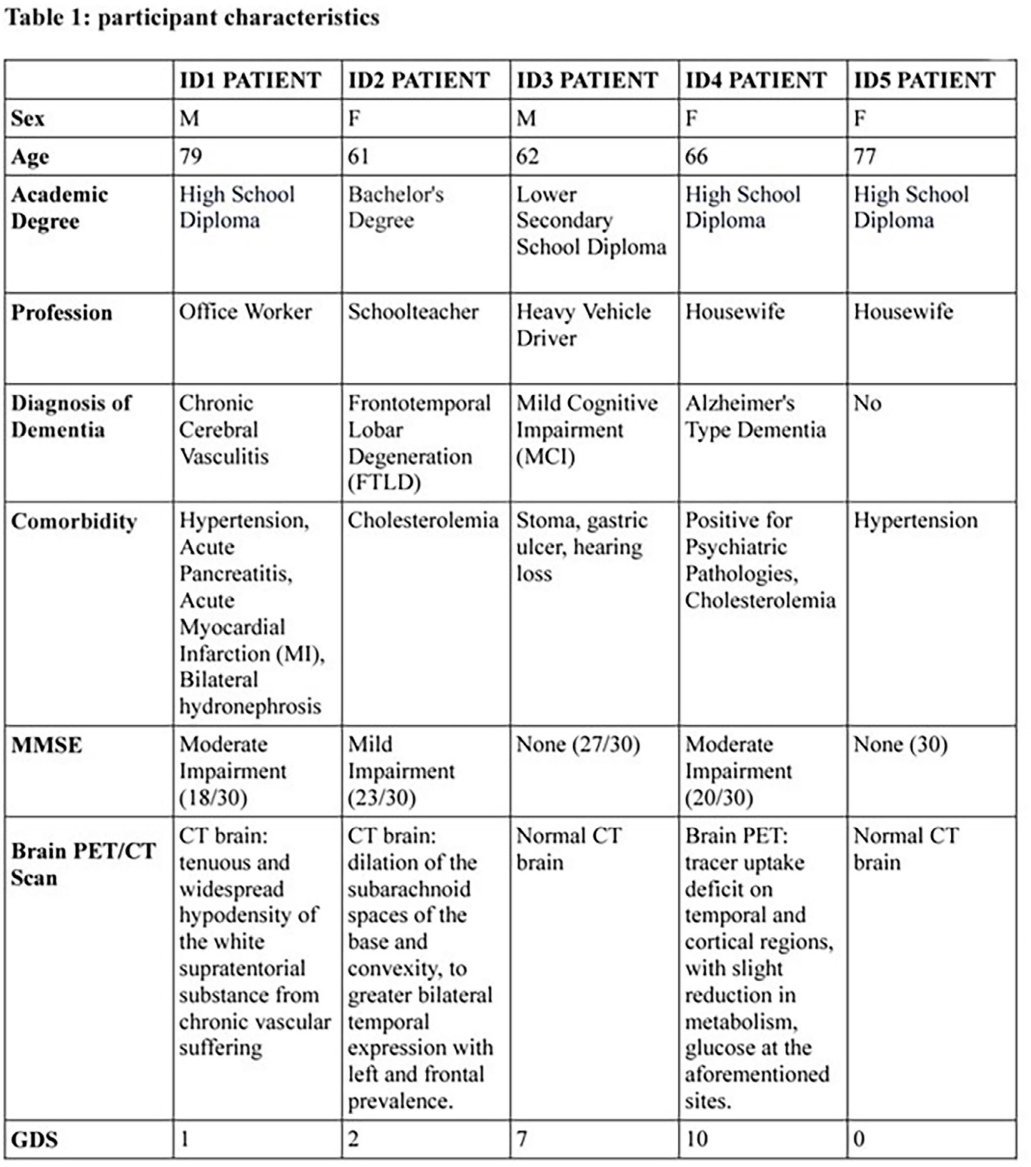# Graphical indices of brain aging

**DOI:** 10.1002/alz.088359

**Published:** 2025-01-09

**Authors:** Carmensita Furlano, Pietro Gareri

**Affiliations:** ^1^ Graphopathologist ‐ CESIOG (Professional study center for training graphologists and writing re‐educators), Cosenza Italy; ^2^ Department of Frailty ‐ Center for Cognitive Disorders and Dementia, Catanzaro Lido, ASP Catanzaro, Catanzaro Italy; ^3^ Center for Cognitive Impairment and Dementia, Catanzaro Italy

## Abstract

**Background:**

Writing is a cortical function studied by the human science of Graphology, which allows the personal knowledge of the subject, and psychophysical conditions, both in healthy older people and in those affected by dementia. Graphologists and geriatricians can successfully cooperate with this.

**Method:**

Five volunteers aged between 60 and 80 years old (3 women and 2 men) were enrolled after signing an informed consent. Each participant underwent multidimensional geriatric evaluation, medical examination, and instrumental exams (Table 1). Each person was asked to write the sentence “I breathe the sweet scent of flowers”, on which Graphological Analysis (in blind) was conducted, based on the evaluation of some variables. Graphology is set up through the neurophysiology of four writing movements: Flexion and Abduction determine the ‘cup’, the Extension prepares the attack of the next letter and its implementation, the Adduction intervenes in the eyelets and in all the gestures of return backwards.

**Result:**

In 4 out of 5 participants, the graphological indices of brain aging were present: slow and heavy writing, a broken, laid or slow rhythm, an uncertain and irregular direction, small superfluous traces, shake, angular and discontinuous movements, the presence of juxtapositions, tremors, disproportions, inequalities in size and deformation of individual letters, tightening of the letters, or high magnification.

**Conclusion:**

It is necessary to promote the presence of the Graphologist in the Healthcare fields, by creating local Geriatric Graphology Centers, in collaboration with multidisciplinary teams, where prevention, diagnosis, rehabilitation/therapeutic treatment, study and research activities can be carried out.